# NMR-based metabolomic techniques identify potential urinary biomarkers for early colorectal cancer detection

**DOI:** 10.18632/oncotarget.22402

**Published:** 2017-11-11

**Authors:** Zhening Wang, Yan Lin, Jiahao Liang, Yao Huang, Changchun Ma, Xingmu Liu, Jurong Yang

**Affiliations:** ^1^ Radiology Department, Second Affiliated Hospital, Shantou University Medical College, Shantou 515041, Guangdong Province, China; ^2^ Radiation Oncology, Affiliated Tumor Hospital, Shantou University Medical College, Shantou 515041, Guangdong Province, China; ^3^ Surgery Department, Second Affiliated Hospital, Shantou University Medical College, Shantou 515041, Guangdong Province, China; ^4^ Shantou University Central Laboratory and NMR Unit, Shantou 515041, Guangdong Province, China

**Keywords:** colorectal cancer, metabolomics, ^1^H NMR spectroscopy, urine, biomarker

## Abstract

Better early detection methods are needed to improve the outcomes of patients with colorectal cancer (CRC). Proton nuclear magnetic resonance spectroscopy (^1^H-NMR), a potential non-invasive early tumor detection method, was used to profile urine metabolites from 55 CRC patients and 40 healthy controls (HCs). Pattern recognition through orthogonal partial least squares-discriminant analysis (OPLS-DA) was applied to ^1^H-NMR processed data. Model specificity was confirmed by comparison with esophageal cancers (EC, n=18). Unique metabolomic profiles distinguished all CRC stages from HC urine samples. A total of 16 potential biomarker metabolites were identified in stage I/II CRC, indicating amino acid metabolism, glycolysis, tricarboxylic acid (TCA) cycle, urea cycle, choline metabolism, and gut microflora metabolism pathway disruptions. Metabolite profiles from early stage CRC and EC patients were also clearly distinguishable, suggesting that upper and lower gastrointestinal cancers have different metabolomic profiles. Our study assessed important metabolomic variations in CRC patient urine samples, provided information complementary to that collected from other biofluid-based metabolomics analyses, and elucidated potential underlying metabolic mechanisms driving CRC. Our results support the utility of NMR-based urinary metabolomics fingerprinting in early diagnosis of CRC.

## INTRODUCTION

Colorectal cancer (CRC) is a major cause of mortality in developing countries, and is the third most commonly diagnosed cancer in men and the second in women [1]. Early stage CRC patients have higher 5-year survival rates than those diagnosed at later stages [2]. Improved early CRC detection methods could reduce patient mortality and improve therapeutic responses and prognoses. Although colonoscopy remains the gold standard for diagnosing precancerous lesions and CRC, this approach is invasive, expensive, and uncomfortable [3], precluding it as a cost effective population-based screening test. Tumor biomarkers, including carcinoembryonic antigen (CEA) and fecal occult blood testing (FOBT), are used clinically, but have relatively low sensitivities and specificities [4–6]. These limitations highlight the need for effective, non-invasive screening tools to facilitate early diagnosis of CRC.

Metabolomics, which investigates global changes in small molecular weight metabolites within a given biological specimen [7–9], is the omics cascade endpoint before phenotype [10]. Metabolomics can be used to assess direct correlations between metabolite and biological phenotype changes. Nuclear magnetic resonance (^1^H-NMR) spectroscopy-based metabolomics methods used in high-throughput studies require only minimal sample preparation to profile a wide range of metabolites [8, 11]. Profiling of metabolic variations in CRC patient tissues has revealed changes in lactate, amino acids, carboxylic acids, fatty acids, and the urea cycle as compared to normal tissues [9, 12]. CRC patient serum metabolite profiles showed abnormal, tumor-associated proline metabolism, glycolysis, fatty acid metabolism, arginine, and oleamide metabolism regulation [13]. Our recent fecal metabolomic study showed nutrient malabsorption, disrupted bacterial ecology, and increased glycolysis and glutaminolysis in CRC patients [14].

Urine is a biofluid commonly used by metabolomics researchers [15], because it is easy to collect in large volumes and may provide diagnostic information for many cancer types [16, 17], including CRC [18]. Biomarkers in urine may be derived from cell apoptosis, glomerular filtration of blood plasma, cell sloughing, epithelial cell secretion of exosomes, and other processes [19]. Diet-derived metabolic markers may also provide important diagnostic clues [20, 21]. Several studies demonstrated a correlation between CRC and perturbed urinary metabolomic profiles [18, 22–24], but none described cancer stage-specific changes. Our study investigated metabolomic profiles in CRC patients at different stages compared to healthy controls (HC), and attempted to identify patients with early stage disease (stage I/II). CRC-specific urinary profiles were confirmed through comparison with an esophageal cancer (EC) cohort.

## RESULTS

### CRC patient urine metabolic profiles

**Figure 1:**
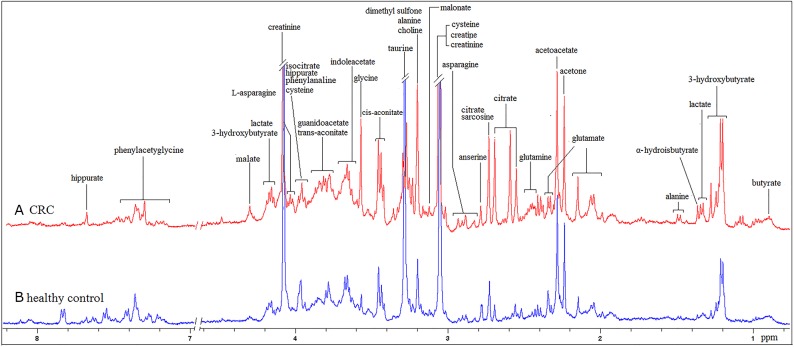
400 MHz representative urine ^1^H NMR spectra from one CRC patient **(A)** and one HC **(B)**, referenced to TSP (0. 0 ppm).

Representative 1D ^1^H-NMR spectra, which provide an overview of all metabolites present in CRC patient and HC urine samples, are shown in Figure 1. Major metabolites were assigned according to previous studies [7, 15] and the Human Metabolome Database (http://www.hmdb.ca/). The aliphatic region at 0.8–4.5 ppm in all spectra included strong signals from water-soluble metabolites, such as lactate, isobutyrate, alanine, glutamate, glutamine, acetone, acetoacetate, creatinine, creatine, cysteine, dimethyl sulfone, malonate, and choline. Isocitrate, hippurate, cysteine, and phenylalanine overlap at 3.96–3.99ppm and are therefore characterized as isocitrate/hippurate/cysteine/phenylalanine in this manuscript.

### Pattern recognition (PR) analyses between disease groups and HCs

**Figure 2:**
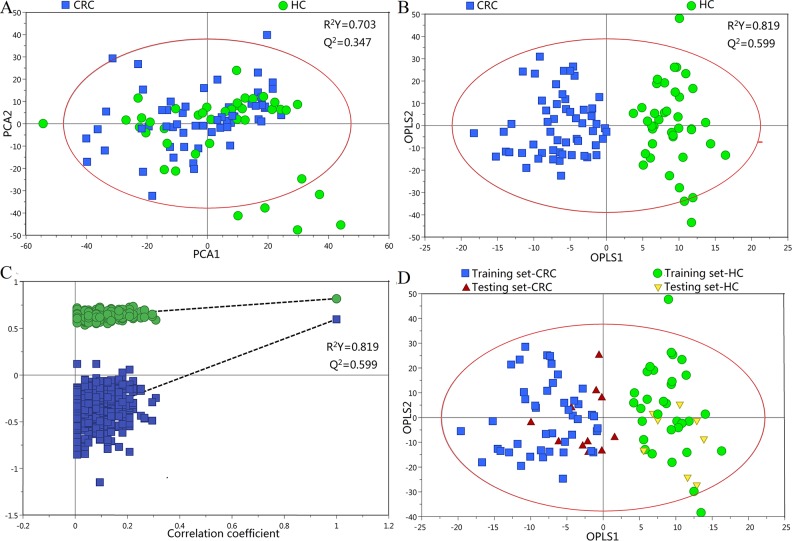
PR of urine metabolomic profiles analyzed using ^1^H-NMR Spectroscopy **(A)** PCA scatter plot of HC (green dots) and CRC patient (blue squares) urine samples. **(B)** OPLS-DA scatter plot based on the same samples. **(C)** Statistical validation of the corresponding OPLS-DA model by permutation analysis (200 times). **(D)** Scores plots of the OPLS-DA prediction model.

High inter-individual variabilities and complexities in urinary profiles made visual comparisons of these spectra impractical. Instead, we used multivariate analysis to determine consistent variations between data sets [7]. Unsupervised PR was initially carried out using preliminary principal component analysis (PCA) to generate an overview of variations between HCs and CRC patients. Clustering based on disease status was not observed on the scores plot of the first two principal components (Figure 2A). A clear separation between CRC patients and HCs was achieved by supervised orthogonal partial least-squares discriminant analysis (OPLS-DA) scores plot (Figure 2B). The internal validation was perfomed to assess the predictive ability of the corresponding OPLS-DA model (R^2^Y=0.819, Q^2^=0.599, CV-ANOVA *p*<0.01), suggesting that the model was a good fit. To futher evaluate the validity of this model, a random permutation test (200 times) was performed, indicating no overfitting (Figure 2C). To assess the predictive ability of the model using unknown samples, 80% of samples (“training set”, HC=32, CRC=45) were randomly selected to construct an OPLS-DA model, which was then used to predict the remaining 20% (“testing set”, HC=8, CRC=10). Testing set HCs were correctly localized to the training set HC region, and equivalent results were obtained using the CRC testing set (Figure 2D).

**Figure 3:**
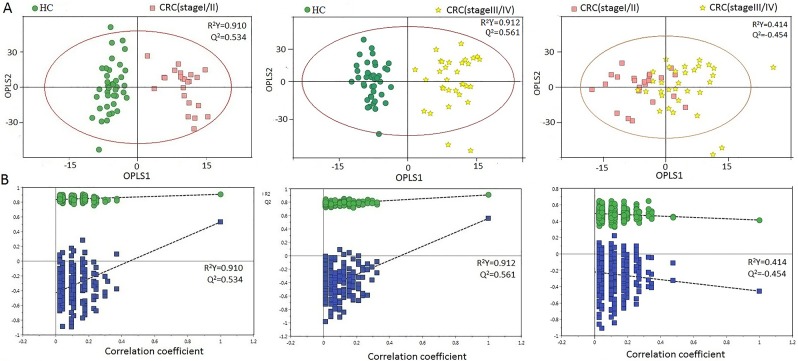
PR analysis of ^1^H-NMR urine spectra from HCs and different CRC stages **(A)** OPLS-DA scatter plot based on HCs and different CRC stages. **(B)** Statistical validation of the corresponding OPLS-DA model by permutation analysis (200 times).

**Figure 4:**
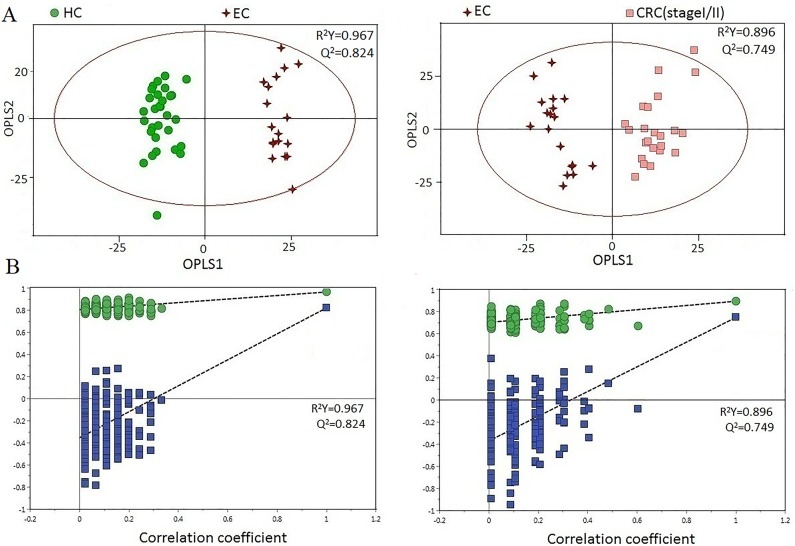
**(A)** OPLS-DA scatter plot based on EC, HC, and stage-I/II CRC samples. **(B)** Statistical validation of the corresponding OPLS-DA model by permutation analysis (200 times).

Urine profiles from HCs and CRC patients at different stages could be clearly separated using the OPLS-DA scores plot (Figure 3A). Model parameters of the permutation analysis of the different groups were as follows: stage I/II vs HC: R^2^Y=0.910, Q^2^=0.534; stage III/IV vs HC: R^2^Y=0.912, Q^2^=0.561 (Figure 3B). Urine metabolite differences between early and advanced stage CRC were less pronounced (two components with R^2^Y=0.414, Q^2^=-0.454). Model specificity was confirmed via comparison with the EC cohort. The OPLS-DA scores plot revealed clear distinctions between early stage CRC, EC, and HC (Figure 4).

### Urinary metabolites contributing to CRC early detection

**Figure 5:**
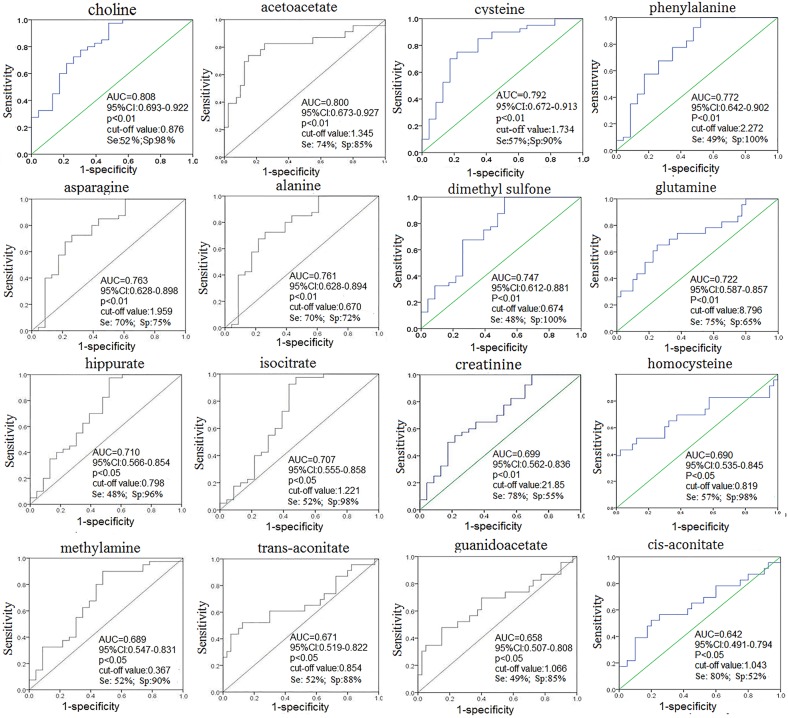
ROC curve of urine metabolites for distinguishing stage I/II CRC patients from HCs

**Figure 6:**
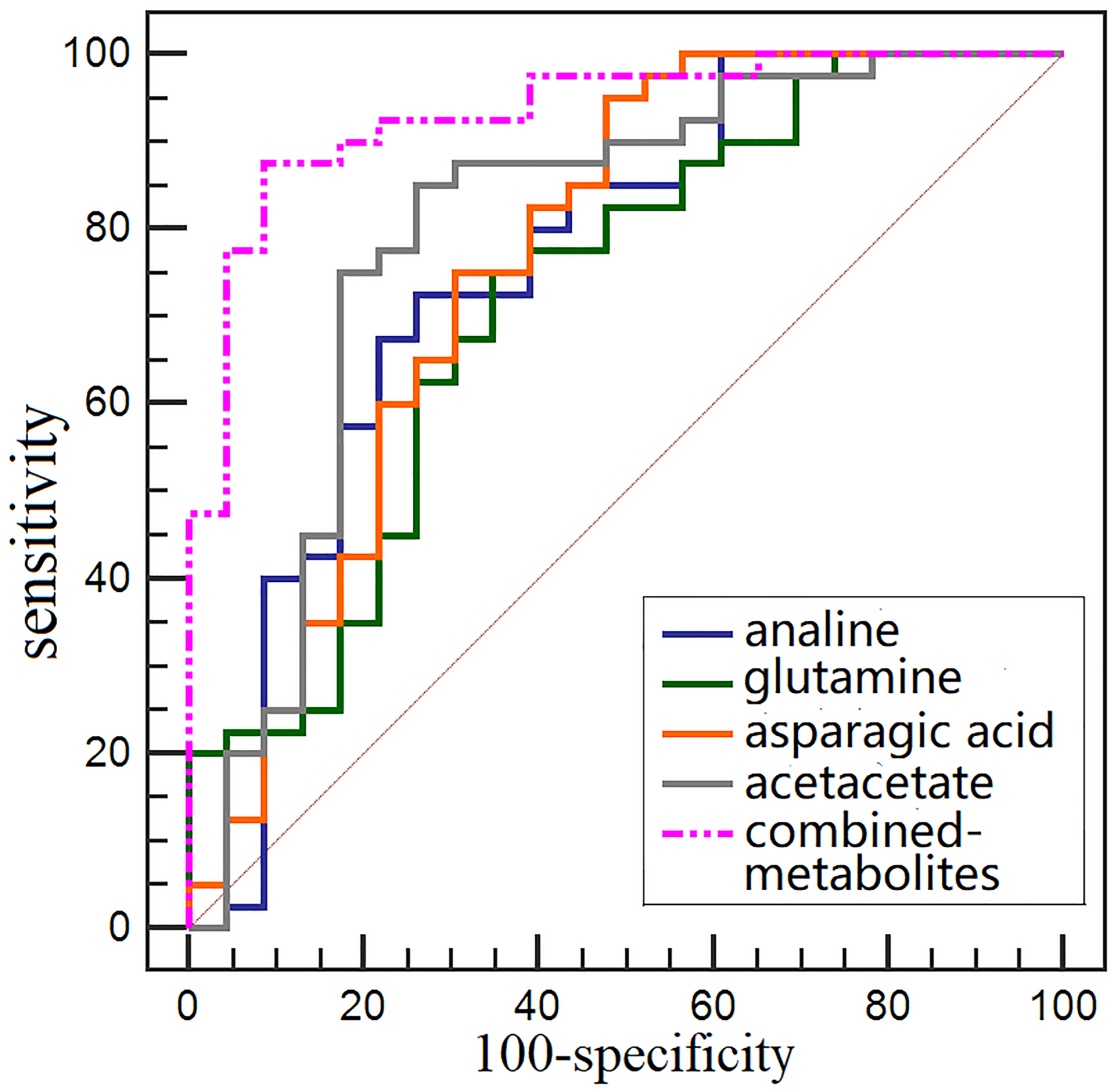
Comparision of single metabolite and combined metabolites ROC curves for distinguishing early stage CRC patients from HCs

We used the following conditions to classify urine metabolites as candidate biomarkers for CRC early detection: (1) metabolites with variable importance in the projection (VIP)>1, and (2) metabolites with differing levels (*p*<0.05 via Mann-Whitney U test) in stage I/II CRC patients vs. HCs. Sixteen of the most significant urine metabolites contributing to patient separation are displayed in Table 1. Among these metabolites, acetoacetate, glutamine, guanidoacetate, cis-aconitate, trans-aconitate, and homocycteine were increased, while creatinine, choline, dimethyl sulfone, asparagine, alanine, methylamine, and isocitrate/hippurate/cysteine/phenylalanine were decreased in the urine of stage I/II CRC patients compared to HCs (*p*<0.01). Optimal cut-off values, sensitivities, specificities, and AUROC values of these metabolites were shown in Figure 5. Acetoacetate, glutamine, asparagine, and cysteine had relatively high AUC values, sensitivities, and specificities in distinguishing early stage CRC patients from HCs. Alanine, glutamine, aspartic acid, and acetoacetate combined had better diagnostic capabilities than any single metabolite alone in discriminating between early stage CRC patients and HCs, with sensitivity, specificity, and AUC values of 87.5%, 91.3%, and 0.933, respectively (Figure 6).

**Table 1:** Marker metabolites found in OPLS-DA models of ^1^H NMR-based urine metabolic profiling between I/II stage of CRC and healthy controls

Top 16 most significant in order	Chemical shift (ppm)	I/II stage of CRC vs healthy controls			Related metabolomic pathway
		VIP^a^	*p*-value^b^	Variations vs HC	
Choline	3.19(s)	2.25	0.000	↓	Choline metabolism, Lipid metabolism
Phenylalanine	3.19(m) 3.98(dd)	2.25 1.43	0.000 0.004	↓ ↓	Amino acid metabolism, Phenylalanine metabolism
Asparagine	2.80(dd)	1.90	0.000	↓	Urea metabolism, Amino acid metabolism
Isocitrate	3.98(d)	1.84	0.005	↓	TCA cycle
Cysteine	3.97(dd) 3.06(m)	1.84 1.70	0.003 0.000	↓ ↓	Amino acid metabolism, Glutamate Metabolism, Glutathione Metabolism
Hippurate	3.96(d)	1.84	0.005	↓	Gut microflora metabolism
Dimethyl sulfone	3.138(s)	1.72	0.000	↓	Gut microflora metabolism, Endogenous methanethiol metabolism
Creatinine	3.03(s) 4.05(s)	1.70 1.43	0.007 0.003	↓ ↓	Urea metabolism, Creatinine metabolism
Alanine	1.46(d)	1.67	0.017	↓	Amino acid metabolism, Gluconeogenesis
Methylamine	2.59(s)	1.12	0.018	↓	Gut microflora metabolism, Disulfiram Pathway
Homocysteine	2.14(m)	1.76	0.014	↑	Amino acid metabolism, Methionine metabolism
Glutamine	2.12(m) 3.76(t)	1.76 1.53	0.001 0.017	↑ ↑	Glutaminolysis, TCA cycle
cis-Aconitate	3.43(d)	1.64	0.043	↑	TCA cycle, Glyoxylate, Dicarboxylate metabolism
Acetoacetate	2.27(s) 3.43(s)	1.59 1.64	0.009 0.043	↑ ↑	Fatty acid metabolism, TCA cycle
trans-Aconitate	3.74(s)	1.54	0.019	↑	TCA cycle
Guanidoacetate	3.78(s)	1.53	0.017	↑	Urea metabolism

a: Variable importance in the projection (VIP) was obtained from OPLS-DA with a threshold of 1.0.

b: *p* value was calculated from Mann-Whitney U test.

### Colorectal cancer-specific metabolomic profiles

Important metabolites for distinguishing CRC from EC patients included the TCA cycle intermediates (fumarate and cis-aconitate), amino acid metabolism (homogentisic acid, indoleacetate), urea metabolism (creatinine, creatine and urea), nucleotide metabolism (thymidine and uracil), gut microflora-derived metabolites (hippurate), glutaminolysis metabolites (glutamine), and others (pyridoxic acid, cinnamic acid, oxypurinol, and trigonelline) (Table 2).

**Table 2:** Marker metabolites found in OPLS-DA models of ^1^H NMR-based urine metabolic profiling between stage I/II CRC patients and EC patients

Metabolites	Chemical shift (ppm)	I/II stage of CRC vs EC			Related metabolomic pathway
		VIP^a^	*p*-value^b^	Variations vs EC	
Thymidine	6.28(t) 7.63(s)	1.82 1.36	0.000 0.033	↑ ↑	Nucleotide metabolism
Fumarate	6.51(s)	1.71	0.000	↑	TCA cycle, Arginine and Proline metabolism, Aspartate metabolism
Hippurate	7.54(m) 7.62(tt)	1.47 1.52	0.036 0.019	↑ ↑	Gut microflora metabolism
cis-Aconitate	6.58(s)	1.69	0.000	↑	TCA cycle, Glyoxylate, Dicarboxylate metabolism
Pyridoxic acid	7.53(s) 4.51(s)	1.62 1.50	0.003 0.000	↑ ↑	Vitamin B6 metabolism
Cinnamic acid	6.51(d) 7.39(d) 7.44(m) 7.61(dd)	1.71 1.31 1.62 1.53	0.000 0.022 0.001 0.019	↑ ↑ ↑ ↑	unknown
Homogentisic acid	6.71(m)	1.23	0.000	↑	Phenylalanine and tyrosine metabolism, Disulfiram pathway
Indoleacetate	7.24(m) 7.50(d) 7.62(d)	1.23 1.47 1.18	0.002 0.009 0.004	↑ ↑	Amino acid metabolism, Tryptophan metabolism
Trigonelline	8.83(m)	1.42	0.043	↑	Vitamin metabolism
Creatinine	3.03(s) 4.05(s)	1.67 1.25	0.000 0.008	↓ ↓	Urea metabolism, Creatinine metabolism
Creatine	3.02(s)	1.67	0.000	↓	Urea metabolism, Creatine metabolism
Uracil	5.79(d)	1.47	0.003	↓	Pyrimidine metabolism
Urea	5.78(br, s)	1.41	0.003	↓	Urea metabolism

a: Variable importance in the projection (VIP) was obtained from OPLS-DA with a threshold of 1.0.

b: *p* value was calculated from Mann-Whitney U test.

## DISCUSSION

Apart from genomic and proteomic alterations, CRC development and progression are associated with cellular metabolic changes that may provide insight into disease pathogenesis [8, 9, 12, 14]. Our ^1^H-NMR-based metabolomic findings identified distinct disturbances to CRC patient urine metabolites, even at stage I/II, compared with HCs, including elevated acetoacetate, guanidoacetate, cis-aconitate, trans-aconitate, glutamine, and homocycteine, and reduced creatinine, phosphorylcholine, dimethyl sulfone, asparagine, alanine, isocitrate, hippurate, methylamine, cysteine, and phenylalanine (Table 1). Altered urine metabolite levels could indicate perturbed amino acid metabolism, glycolysis, TCA cycle, urea cycle, choline metabolism, and gut microflora metabolism. OPLS-DA differentiated metabolic profiles from stage I/II CRC, EC, and HC, indicating that upper and lower gastrointestinal cancers have different metabolomic profiles [25]. Our study assessed important metabolomic variations in CRC patient vs. EC patient and HC urine samples, providing information complementary to that derived from other biofluid-based metabolomics studies, and adding to our understanding of the metabolic mechanisms driving CRC.

While many metabolites that differ between cancer patients and HCs have diagnostic potential, only those with potential biological relevance are of practical use. Choline was the most significantly altered metabolite, with lower levels and higher VIP values in cancer patients. Lower urinary choline levels in CRC are most likely related to increased demand for choline in tumors. Choline contributes to tumor cell phospholipid synthesis, and is integrated into lecithin, a major cell membrane phospholipid component [26]. Consistent with a previous report, as choline demand increases in tumor tissues, blood choline levels drop, leading to decreased urinary choline in CRC patients [27]. Another plausible explanation for decreased urinary choline levels might be its utilization as an alternative methyl group donor for DNA methylation and synthesis in tumor cells [28]. Additionally, levels of the choline precursor, creatinine, were decreased in CRC patient urine samples [29]. Our findings suggest that choline could be a viable biomarker associated with tumor promotion.

**Figure 7:**
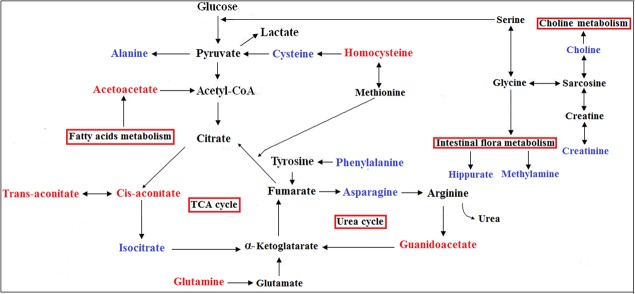
Metabolic pathways that include the most promising potential CRC metabolite biomarkers identified in this study Red represents metabolites upregulated in CRC, blue represents those downregulated.

Another key altered metabolite is isocitrate, an important intermediate in the TCA cycle (Figure 7). Isocitrate levels were reduced in CRC patient urine compared to that of HCs, suggesting TCA cycle deregulation and increased energy metabolism due to tumor cell activation [30, 31]. Isocitrate can be converted to citrate and α-ketoglutarate, and this process is balanced to allow generation of both ATP and cellular macromolecules to sustain cell growth. Since glucose is the main carbohydrate source for glycolysis and the TCA cycle, increased glycolysis, as previously reported in CRC patient tissue, serum, and fecal metabolomic studies, may also lead to reduced TCA intermediates in urine [12, 14, 32]. However, we observed slightly higher levels of cis-aconitate, a TCA cycle intermediate produced by the dehydration of citrate, in CRC patient urine. This could result from elevation of trans-aconitate, which is then converted into cis-aconitate (Figure 7). Acetoacetate, a catabolite of fatty acids metabolism during calorie restriction, was upregulated in CRC patient urine compared with that of HCs, consistent with increased energy consumption by the tumor. Acetoacetate upregulation leads to increased production of acetyl-CoA, an intermediary that promotes TCA cycle alternative energy utilization in cancers when glucose and TCA intermediates (such as isocitrate) are insufficient [33]. Increased glutamine levels were also observed in early stage CRC urine samples, suggesting augmented glutaminalysis. Glutamine is lysed to glutamate, which can be converted to α-ketoglutarate to increase transit through the citric acid cycle, providing sustainable energy required for rapid cell proliferation [34, 35]. Decreased asparagine with an equivalent increase in guanidoacetate levels observed in CRC urine samples might result from increased urea cycle activity to maintain urea detoxification. Compared to HCs, increased homocysteine and decreased alanine, cysteine, and phenylalanine were measured in CRC patient urine. This could be accounted for by disrupted amino acid metabolism due to epithelium inflammation and injury resulting from bowel disease in CRC patients [36]. The observed depletion of methylamine and hippurate in urine suggests a disruption in the intestinal epithelium and diffusion of gut microbes associated with colorectal tumourigenesis [37]. Among the potential urinary biomarkers, acetoacetate, glutamine, asparagine, and cysteine had relatively high AUC, sensitivity, and specificity values for distinguishing early stage CRC patients from HCs. However, the diagnostic performance of analine, glutamine, aspartic acid, and acetoacetate combined improved discrimination between early stage CRC patients and HCs compared to any single metabolite alone.

To determine whether the urinary metabolic alterations observed here were specific to CRC or reflective of common metabolic changes associated with malignancy, we compared the metabolomic profiles of patients with CRC and EC. We identified key discriminatory metabolites through VIP analysis, which revealed several distinguishing patterns. We also observed overlapping metabolites between CRC and EC, which could reflect metabolic changes associated with shared tumorigenesis pathways, including disturbed gut microflora metabolism and urea metabolism associated with tumor cell proliferation and growth.

Overall, our findings revealed that CRC patient urinary metabolic profiles can be distinguished from those of HCs, even in early stages of disease (stage I/II), supporting the utility of NMR-based urinary metabolomics fingerprinting in early diagnosis of CRC. Our results demonstrate the potential of this noninvasive urinary metabolomic strategy as a complementary diagnostic tool to screen for early CRC tumor-associated metabolic pathway perturbations. While our preliminary results are encouraging, the presence of a given metabolite in urine is the result of complex production, utilization, and glomerular filtration pathways. Therefore, further investigations must be undertaken to assess and validate each metabolite with biomarker potential identified in our study.

## MATERIALS AND METHODS

### Patient recruitment and sample collection

This study was approved by the Ethics Committee of Shantou University Medical College. Written informed consent was obtained from each subject prior to participation in the study. Preoperative, early-morning midstream urine samples were collected from patients with CRC (n=55) or EC (n=18), and from HCs (n=40), between January and July 2015 at the Second Affiliated Hospital of Shantou University Medical College. Patients did not receive any neoadjuvant chemotherapy or radiation therapy prior to sample collection. Patient samples were categorized according to histopathological features. For all cases, histologic findings were obtained and follow-up data were available to ensure accurate disease classification. No cancer patients had complicating diseases. Exclusion criteria were: breastfeeding, pregnancy, inflammatory conditions, gastrointestinal tract disorder, mental disorder, hypertension, diabetes mellitus, other metabolic diseases, and urinary tract involvement, such as uncontrolled bacterial, viral, or fungal infection,. Healthy controls were age- and gender-matched patients, and had no declared history of cancer or gastrointestinal symptoms. Patient demographic and clinical characteristics are summarized in Table 3.

**Table 3:** Summary of clinical and demographic features for study subjects and tumor characteristics

	CRC (55)			EC (18)	HC (40)
	Stage-I/II	Stage-III/IV	*p* value		
Age (median, range)	61, 27-84	59, 38-81		61, 32-77	59, 28-78
Sex			0.944		
Male	11	15		8	19
Female	12	17		10	21
Cancer site					
Colon	10	23	0.034		-
Rectum	13	9			-
Cervical				2	-
Upper Thoracic				3	-
Middle Thoracic				5	-
Lower Thoracic				8	-
FOBT			0.767		
Positive	9	14		N/A	N/A
Negative	1	3		N/A	N/A
Not check	13	15			
CEA (ng/mL)			0.029		
Positive	5	18		5	N/A
Negative	15	11		3	N/A
Not check	3	3			
CA199(U/mL)			0.563		
Positive	3	8		3	N/A
Negative	15	17		4	N/A
Not check	5	7			
Tumor size (cm, Mean ± SD)					
Major diameter	5.14±3.28	5.38±1.71	0.756		
Width diameter	3.90±2.08	4.21±1.52	0.573		
Thick diameter	1.92±1.14	2.38±1.21	0.119		
Symptoms			0.184		
Change of stool character	10	18			
Hematochezia	12	10			
Abdominal pain	8	9			
Bowel obstruction	3	2			
Weight			0.779		
<50kg	5	8			
≥50kg	18	24			

### Urine sample preparation

The preservative, sodium azide (50 μL), was added to each urine sample before storing at −80°C. Frozen urine samples were thawed at room temperature and mixed to suspend any settled precipitate. Then, 300 μl PB/D_2_O buffer (0.1 M, pH=7.4) was added to 600 μl of each sample, and the mixture was vortexed and then centrifuged at 8,000 rpm for 10 min. Finally, a stock solution of sodium (3-trimethylsilyl)-2,2,3,3-tetradeuteriopropionate (TSP)/D_2_O (50 μL) was added to each supernatant prior to analysis via ^1^H NMR spectroscopy.

### ^1^H NMR spectroscopy

Urine **^1^**H NMR spectra were obtained on a Bruker AVII 400.13 MHz ^1^H-NMR spectrometer (Bruker Biospin, Germany) using a one-dimensional NOESY (nuclear overhauser enhancement spectroscopy) pulse sequence [RD-90°-*t_1_*-90°-*t_m_*-90°-ACQ] with the following acquisition parameters: recycle delay, RD=1.5 s; *t_1_*=3 μs; mixing time, *t_m_*=100 ms; 90° pulse width=7.3 μs; number of scans, NS=256; number of points, TD=32768; spectral width, SW=8012.82 Hz; acquisition time, AQ=2.04 s. Water suppression was achieved via irradiation of the water peak during RD and *t_m_*.

### ^1^H NMR spectral data processing

All **^1^**H NMR spectra were multiplied by a 0.3 Hz exponential line broadening prior to Fourier Transformation, and then were corrected for phase and baseline distortion and calibrated to TSP at 0.0 ppm. ^1^H-NMR data complexity was reduced through segmentation of the spectral range from 0.5–9.0 ppm with the equal width of 0.004 ppm. The imperfect water signal from 5.5–4.5 ppm was discarded, and each bucket was internally normalized to the total integral of the spectrum prior to pattern recognition.

### Pattern recognition (PR) analysis and cross validation

To establish a global overview of differences between CRC and EC patients and between CRC patients and HCs, a multivariate analysis was applied to **^1^**H NMR spectra data as previously described [14]. Normalized ^1^H NMR spectral data sets were unit variance scaled, and then analyzed using the SIMCA-P+ program (version 14.1, Umetrics AB; Umeå, Sweden). A PCA model was applied to the mean-centered, normalized **^1^**H NMR spectra to detect general trends and outliers, followed by OPLS-DA. Model quality was evaluated using R**^2^**Y and Q**^2^** values, which reflect the explained fraction of variance and model predictability. VIP values of all peaks from OPLS-DA models were taken as a coefficient for peak selection. VIP was represented by a unitless number; the higher the value, the greater the discriminatory power of the metabolite. Those variables with VIP>1 were considered potential biomarker candidates for group discrimination.

### Statistical analysis

Relative concentrations of those metabolites with VIP>1 were calculated by integrating the signals in the spectra. Differences between stage I/II CRC patients and HCs were assessed using the Mann-Whitney U test and, *p*<0.05 was considered statistically significant. Receiver operating characteristic (ROC) analysis was performed in SPSS 16.0 to further evaluate the diagnostic power of potential biomarkers. The area under the ROC curve (AUROC), specificity, sensitivity, and accuracy of the metabolites were calculated, where AUROC>0.8 indicated excellent diagnostic performance.
